# Data quality assessments stimulate improvements to health management information systems: evidence from five African countries

**DOI:** 10.7189/jogh.09.010806

**Published:** 2019-06

**Authors:** Jennifer Yourkavitch, Debra Prosnitz, Samantha Herrera

**Affiliations:** 1 ICF, Rockville, Maryland, USA; 2 Save the Children U.S., Washington, D.C., USA

## Abstract

**Background:**

Health service data are used to inform decisions about planning and implementation, as well as to evaluate performance and outcomes, and the quality of those data are important. Data quality assessments (DQA) afford the opportunity to collect information about health service data. Through its Rapid Access Expansion Programme (RAcE), the World Health Organization (WHO) funded non-governmental organizations (NGO) to support Ministries of Health (MOH) in implementing integrated community case management (iCCM) programs in the Democratic Republic of Congo, Malawi, Mozambique, Niger and Nigeria. WHO contracted ICF to support grantee monitoring and evaluation efforts, part of which was to conduct DQAs to enhance program monitoring and decision making. The contribution of DQAs to data-driven decision making has been documented and the purpose of this paper is to describe how DQAs contributed to health management information system (HMIS) strengthening and the findings of subsequent DQAs in those areas.

**Methods:**

ICF created a mixed-methods DQA for iCCM data, comprising a review of the data collection and management system, a data tracing component and key informant interviews. The DQA was applied twice in each RAcE site, which enables a general comparison of system-level attributes before and after the first DQA application. For this qualitative assessment, we reviewed DQA reports to collate information about DQA recommendations and how they were addressed before a subsequent DQA, along with the findings of the second DQA.

**Results:**

Findings from the first DQA in each RAcE site stimulated NGO and MOH efforts to strengthen different aspects of the HMIS in each country, including modifying data collection tools in the Democratic Republic of Congo; training community health workers (CHWs) and supervisors in Malawi; strengthening supervision in Mozambique; improving CHW registers and strengthening staff capacity at all levels to report data in Niger; establishing a data review system in Abia State, Nigeria; and, establishing processes to improve data use and quality in Niger State, Nigeria.

**Conclusion:**

Data quality assessments stimulated context-specific efforts by NGOs and MOHs to improve iCCM data quality. DQAs can serve as a collaborative and evidence-based activity to influence discussions of data quality and stimulate HMIS strengthening efforts.

Given that health service data are used to inform decisions about planning and implementation, as well as to evaluate performance and outcomes, the quality of those data are important. Data quality assessments (DQA) afford the opportunity to collect information about health service data and to develop a profile of data quality, evaluating characteristics including accuracy, consistency, and the timeliness of data reports. DQAs can also provide information about the health management information system (HMIS) that may support or hinder data quality. The World Health Organization (WHO) and partners recently released a three-part manual to aid assessments of data quality, underscoring growing global interest in this topic [1].

Through its Rapid Access Expansion Programme (hereafter referred to as RAcE), the WHO funded non-governmental organizations (NGO) to support Ministries of Health (MOH) in implementing integrated community case management (iCCM) programs in the Democratic Republic of Congo, Malawi, Mozambique, Niger, and Nigeria. WHO contracted ICF to support grantee monitoring and evaluation (M&E) efforts, part of which was to conduct DQAs to enhance program monitoring and decision making. Grantee data were sourced from the iCCM data reported by community health workers (CHWs) and MOH staff in communities and facilities, so the DQAs were able to assess the quality of iCCM data and relevant aspects of the HMIS in each country. Results from the assessments informed recommendations to improve data collection and management.

The potential contribution of DQAs to data-driven decision making has been implied elsewhere [2-5]. The purpose of this paper is to describe how DQAs contributed to HMIS strengthening, specifically, the recommendations yielded by DQAs in each RAcE-supported programme area, the HMIS improvement efforts undertaken following the DQA, and the findings of subsequent DQAs in those areas.

## METHODS

ICF created a mixed-methods DQA for iCCM data, described in detail elsewhere [6]. The assessment comprised a review of the data collection and management system at all levels (community, facility, district, and central), a component that traced data reported between levels, and key informant interviews. The DQA was applied twice in each RAcE site, which enables a general comparison of system-level attributes before and after the first DQA application. ICF obtained ethical approval from ICF’s Institutional Review Board as well as from institutions in each country before conducting each DQA. This thematic meta-analysis of those DQAs does not constitute human subjects research.

**Figure 1:**
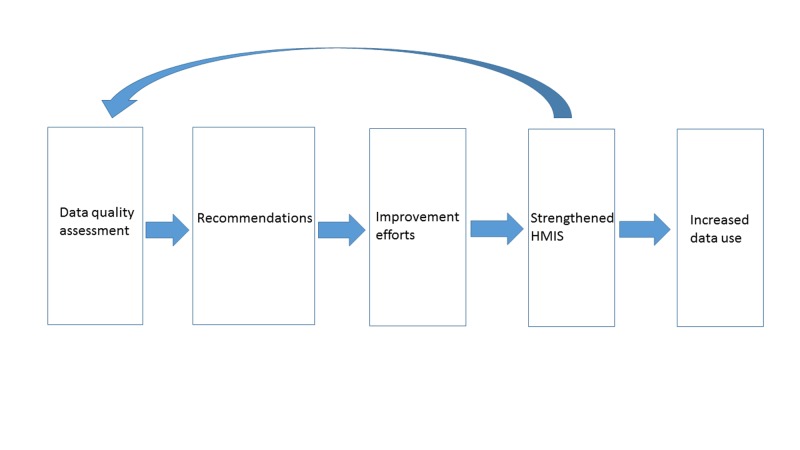
Theory of change for Data quality assessments (DQAs) and Health Management Information System strengthening.

For this qualitative assessment, we reviewed DQA reports to collate information about DQA recommendations and how they were addressed before a subsequent DQA, along with the findings of the second DQA. **Figure 1** illustrates a simple post-hoc theory of change for this analysis and a continuous improvement cycle that could be engendered with multiple DQAs.

We listed and compared system-wide assessment results between the two DQAs in each country. The system-level assessment measured data quality factors in these five domains: Monitoring and Evaluation Structure, Functions and Capabilities; Indicator Definitions, and Reporting Guidelines; Data Collection and Reporting Forms and Tools; Data Management Processes; and Links with the National Reporting System. We scored a series of indicators in each domain on a scale (1 = Low; 2 = Moderate; 3 = Strong) and then calculated an average score per domain. Online Resource 1 lists the indicators in each domain of the systems assessment of the DQA. We reported a relative, qualitative value for each DQA’s findings in these descriptions and efforts to strengthen the HMIS in response to recommendations from the first DQA. We focused this qualitative, thematic analysis on a general comparison of findings between the two DQAs.

## RESULTS

The average interval between DQAs was 15 months. DQA timing depended on ICF, NGO, and MOH staff availability. While the short timeframe likely had an impact on the type of improvements that could be made (eg, “low-hanging fruit”), nonetheless, there is evidence that findings from the first DQA in each RAcE site stimulated grantee and MOH efforts to strengthen different aspects of the HMIS in each country (**Table 1**). A summary description of findings from this analysis is presented here.

**Table 1:** Summary of DQA recommendations, improvement efforts, and findings

Country and first DQA year	Recommendations	Improvement efforts	Second DQA year and findings
**Democratic Republic of Congo, 2014**	-Address the weaknesses of the monitoring system, especially the duplication of reporting -Address the complexity of data collection tools -Translate tools into the local language of CHWs -Bind reporting forms for easier maintenance and storage *[Domains: Indicator Definitions and Reporting Guidelines, and Data Management Processes]*	Modified data collection tools	**2015 positive findings:** -Stronger results in all but one domain -CHWs were comfortable using reporting forms **Negative findings:** -No written guidelines for completing data reporting forms -Completing reporting forms is time consuming for CHWs
**Malawi, 2014**	-Standardize data reporting procedures -Address late reports, missing and implausible values, and incorrect aggregation -Conduct refresher trainings for CHWs and their supervisors *[Domains: Indicator Definitions and Reporting Guidelines, and Data Management Processes]*	-CHW and supervisor trainings on data collection and reporting -Job aid created to guide CHW reporting	**2016 positive findings:** -High scores in each domain -Trainings for CHWs and supervisors were conducted **Negative findings:** -Limited capacity in data management processes -No written reporting guidelines for district and central level -Supervisors did not spend much time on data quality -Lack of controls to prevent double counting and to identify people who do not follow referrals
**Mozambique, 2015**	Strengthen the reporting capacity of CHWs and supervisors Strengthen the supervision system Create standard protocols throughout the reporting system *[All domains were moderate or high.]*	M&E manual was updated to include information about managing data	**2016 positive findings:** iCCM data reporting system is well-established; all but one domain scored highly Regular CHW supervision visits occurred **Negative findings:** No process to track referrals No process to avoid double counting No systematic documentation of how data discrepancies were resolved
**Niger, 2014**	-Standardize and simplify CHW registers -Strengthen supervision by using a checklist -Provide written data management protocols for all levels of the reporting system *[Domains: Indicator Definitions and Reporting Guidance, Data Management Processes, and Links with the National Reporting System.]*	-Staff at all levels were trained on data reporting -Data collection process streamlined so that indicator points could be found on forms at each level -Registers revised to facilitate completion	**2015 positive findings:** -System assessment yielded moderate scores **Negative findings:** -Some supervisors did not have time for supervision -Reporting forms occasionally unavailable for CHWs -Could not integrate iCCM data with HMIS due to HMIS updating process
**Nigeria (Abia State), 2015**	-Create a data governance structure to clearly establish roles and responsibilities for iCCM data reporting at all levels of the system -Develop data reporting and management procedures at all levels and guidelines for how to complete reporting forms Improve regular supervision Hold data review meetings and promote iCCM data use *[Domains: Indicator Definitions and Reporting Guidance, Data Management Processes, and Links with the National Reporting System.]*	-Refresher trainings for CHWs and supervisors -Development of an LGA summary reporting form to aggregate data from CHEWs and promote supervision of CHEWs at the LGA level -Held regular data review meetings	**2016 positive findings:** -High scores for all but one domain **Negative findings:** -Errors in aggregated data at supervisors’ level -Written guidance for data reporting not available -iCCM data integration into HMIS under discussion
**Nigeria (Niger State), 2015**	-Organize additional trainings for CHWs -Hold data review meetings -Strengthen supervision -Create standard data management procedures *[Domain: Indicator Definitions and Reporting Guidelines]*	-Refresher training for supervisors -Processes to improve data use and quality established	**2016 positive findings:** Moderate scores Good understanding about iCCM data reporting at all levels well-established supervision system **Negative findings:** Data not used by CHWs or supervisors No written guidance for reporting data Data management responsibilities not well defined at upper levels of system

DQA – data quality assessment, CHW – community health worker, CHEW – community health extension worker, iCCM – integrated community case management, M&E – monitoring and evaluation, LGA – local government area

### The Democratic Republic of Congo

In the first DQA (2014), two domains scored lower than others: Indicator Definitions and Reporting Guidelines (moderately low) and Data Management Processes (very low). Regarding reporting, CHWs had seven forms to complete in a complex reporting process and their training seemed inadequate for the expectations of their service. There were no written guidelines for completing reporting forms or for aggregating and analyzing data at different levels of the health system. Written procedures for processing overdue or incomplete reports were not available at any level. Recommendations were to simplify the tools, translate the tools into the local language of CHWs, and bind reporting forms for easier maintenance and storage by CHWs. Recommendations also addressed weaknesses of the monitoring system including duplication of reporting at the health area level, and, specifically, holding refresher trainings for all staff involved in data collection.

In 2015, the system assessment showed stronger results in every area except the “indicator definitions and reporting guidelines” domain. Data collection tools had been modified and CHWs were comfortable using them although they reported that completing them was time consuming. There were still no written guidelines for completing forms. Recommendations included continuing to reinforce accurate reporting and extending the CHW pre-service training time devoted to data recording and reporting.

### Malawi

In the first DQA (2014), two domains scored lower than others: Indicator Definitions and Reporting Guidelines (moderately low) and Data Management Processes (very low). Recommendations from that assessment focused on standardizing data reporting procedures, addressing late reports, missing and implausible values, and incorrect aggregation, and conducting refresher trainings for CHWs and their supervisors.

The second DQA (2016) yielded high scores in each domain; however, it also revealed several more areas for improvement. CHWs and their supervisors had been trained on data collection and reporting, and a new job aid in the register improved reporting guidance. However, CHWs had not been trained on data management. In addition, supervisors did not spend much time on checking data quality, as compared to checking performance of duties and supply stock. There were still no written guidelines on data reporting at central and district levels. Although supervision improved for CHWs that had poor data reporting, feedback to CHW supervisors about data reporting errors was not documented systematically and intervals between supervisory visits increased due to the number of CHWs to be visited. In addition, there remained a lack of controls for preventing double counting in the system and for identifying people who do not follow referrals. DHIS2 was introduced between the DQAs and that likely changed the reporting process in some ways.

### Mozambique

The first DQA in Mozambique (2015) scored the system as “moderate” in all areas except for links with the Links with National Reporting System, which scored highly. Recommendations included: strengthening the reporting capacity of CHWs and supervisors; strengthening the supervision system; and, creating standard protocols throughout the reporting system, including how to handle late submission of reports, missing values, incorrect aggregation, and implausible values.

The second DQA in 2016 found that the iCCM reporting system is well established and harmonized with the national reporting system, with designated staff at all levels to aggregate and review data. In addition, CHW reporting was found to be more consistent in this DQA. All domains scored highly except for data management processes. The M&E manual had been updated to include information about managing data after the first DQA. Supervision visits to CHWs occurred regularly. Additional supervision provided via phone or email was also implemented; however it was not documented. The main outstanding issues were the inability to track referrals and a lack of quality controls to avoid double-counting. In addition, there was no systematic documentation of how data discrepancies were resolved.

### Niger

Part of the first DQA in Niger in 2014 could not be conducted due to incomplete CHW registers. Recommendations included standardizing and simplifying CHW registers, strengthening supervision by using a checklist, providing written data management protocols for all levels of the reporting system, and encouraging data use.

By the time of the second DQA in 2015, the quality of data in CHW registers had improved and the system assessment yielded moderate scores. Staff at all levels had been trained on data reporting and had a clear understanding of their roles in data reporting. The data collection process was streamlined after the first DQA, and the indicator data points reported in service delivery registers could be reported on forms to the next system level. Registers had been revised with pre-printed labels and more time was given to record keeping during CHW training. Nonetheless, challenges remained. Some supervisors did not have time for supervision duties, and reporting forms were occasionally unavailable for CHWs. There were challenges with integrating iCCM data into the HMIS because the HMIS was being updated. When CHWs felt overwhelmed with their work they could not prioritize data reporting.

### Nigeria, Abia State

The first DQA in Abia State, Nigeria (2015) scored the system highly in “M&E Structure” and “Data Collection and Reporting Forms and Tools,” but the other domains scored low, particularly “indicator definitions and reporting guidance.” Recommendations included creating a data governance structure with clarified roles and responsibilities at all levels of the iCCM data reporting system, developing data reporting and management procedures at all levels, developing guidelines for completing the reporting forms, providing regular supervision, and promoting review and use of iCCM data through data review and feedback sessions.

The second DQA in 2016 showed high scores for all domains except “Indicator Definitions and Reporting Guidelines.” Refresher trainings had been offered to CHWs and their supervisors. A summary form was developed and implemented at the local government area (LGA) level to improve data flow through the system and promote supervision at the LGA level. Regular data review meetings were implemented. However, some challenges remained. Errors were found after aggregation, suggesting that supervisors needed more training. Written guidelines for data reporting were not available at most system levels. At the time of the DQA, the state was in the process of determining which iCCM indicators would be included in the HMIS.

### Nigeria, Niger State

The first DQA in Niger State, Nigeria in 2015 scored the system as “moderate” in all domains except “Indicator Definitions and Reporting Guidelines,” which scored low. Recommendations included additional trainings for CHWs, holding data review meetings, strengthening supervision, and creating standard data management procedures.

The second DQA (2016) also yielded a moderate score for the “Reporting and Data Management System,” but was weak in the “Link with National Reporting System” and “Indicator Definitions and Reporting Guidelines” domains. The DQA found a good understanding among staff at all levels of their roles in iCCM data collection and reporting; a well-established supervision system including joint supervision by local government areas, the State Ministry of Health, and grantee staff; and the use of national iCCM forms for data collection. There had been a refresher training for supervisors, and processes had been established to improve data use and quality including data display posters and supervision tools. However, iCCM data were not yet included in the HMIS at the State level, nor were there any written guidance for reporting data. In addition, responsibilities for data management were not defined well at upper levels of the system.

## DISCUSSION

These examples indicate the utility of DQAs for stimulating HMIS improvements. Through support from RAcE, efforts were undertaken to improve data reporting systems and data quality between the DQAs in each area. While challenges remained, improvements in system capacity to support high quality data collection and reporting were evident at the time of the second DQAs.

Certainly, the ability of a health system to conduct DQAs, let alone act on the recommendations they produce, is dependent on the resources at hand. Programme resources are usually devoted to all that implementation requires, including training, supervision, and supplies, among other costs. Supervisors were tasked with both reviewing quality of care and quality of data, and with limited time often prioritized reviewing service delivery over data reporting. Rarely are resources devoted to system improvements to support data quality even though national and global policy makers and stakeholders rely on those data to direct further resource investment [6]. Areas supported by RAcE were uniquely positioned to benefit not only from iCCM programmes but also from strengthened data management systems. We presented evidence of health system strengthening efforts through (re)training staff and simplifying and streamlining data reporting. Future studies could attempt to link data quality to quality of care and system strengthening efforts supporting both.

RAcE-supported programmes had different histories with iCCM, eg, at the extremes, Malawi had been implementing an iCCM programme for eight years before RAcE started, while RAcE introduced iCCM for the first time in Nigeria. Thus, there were more challenges with establishing data management systems in Nigeria than there were in Malawi. But there was also an opportunity to instill an appreciation for data quality at all levels of the system there, even as the iCCM programme itself was being established. While in Malawi, challenges remained with ensuring adequate supervision for data quality. The service and associated reporting were not new but the need for some refinement to ensure data quality persisted.

Where the domain of data management processes had been found lacking in the first round of DQAs, this domain was generally found to be improved in the second round. However, the domain of “indicator definitions and reporting guidelines” remained challenging for most areas, in part because there were no written protocols or guidance for data reporting. Establishing these protocols would require leadership from the central level and a comprehensive dissemination strategy. Central level action on this topic may require additional advocacy and deserves internal and external investment. While some recommendations focused on central level leadership, the majority addressed practical issues at the CHW and facility levels. Increased use of data for decision making could lead naturally to greater attention to the quality of those data. Disseminating data reporting protocols throughout the health system could be accomplished through meetings at administrative levels and refresher trainings at service delivery levels. In addition, supervision protocols could incorporate attention to data quality directly.

There are some limitations to this analysis. In some cases, the assessments in each country were led by different staff and the scoring of consecutive assessments may differ, in part, for that reason. However, we focused here on qualitative evidence of HMIS strengthening activities, which should be free from interpretive bias. In addition, we likely did not report all system improvement efforts here. A lesson learned from the experiences of conducting two DQAs in each project area is the importance of documenting efforts to act on the first set of recommendations and linking that documentation to the second DQA report, so that there is a continuous record concerning data quality and related improvement efforts in a HMIS. Finally, the tool itself may be more suited to assessing the basics of a data management system rather than detailed refinements. We found that countries with systems needing basic supports scored lower than countries with established systems, but findings from the latter indicated significant room for improvement nonetheless. In addition, this DQA tool did not focus on data use, although that is clearly a concern (eg, see information presented about Abia State). Future efforts to assess data quality could specify a component related to data use, which is the ultimate purpose of a data system.

## CONCLUSION

The DQAs conducted by ICF stimulated some efforts by NGOs and MOHs to improve iCCM data quality. Improvements were context-specific, but generally included the strengthening of staff skills, which is logically the place to begin data quality improvements. However, efforts to strengthen staff skills require system support to promote sustainable improvement. In addition to trainings, NGOs worked with MOHs to standardize or simplify reporting procedures. The importance of data quality for understanding health service performance and access to and equity in service delivery is clear, and additional resources are needed to improve data quality through system investments that support consistent data reporting. DQAs can serve as a collaborative and evidence-based activity to influence discussions of data quality and stimulate HMIS strengthening efforts.
